# *In Silico* Structural Analysis of the Grapevine Serine Protease VviSBT4.19 Involved in Defense against *P. viticola*

**DOI:** 10.1021/acs.jafc.5c11183

**Published:** 2025-12-24

**Authors:** Filipe E. P. Rodrigues, Sara G. F. Ferreira, Catarina Paiva-Silva, Andreia Figueiredo, Rita B. Santos, Miguel Machuqueiro

**Affiliations:** † BioISI - Instituto de Biossistemas e Ciências Integrativas, Departamento de Química e Bioquímica, 111161 Faculdade de Ciências, Universidade de Lisboa, 1749-016 Lisboa, Portugal; ‡ Grapevine Pathogen Systems Lab, BioISI - Instituto de Biossistemas e Ciências Integrativas, Faculdade de Ciências, Universidade de Lisboa, 1749-016 Lisboa, Portugal

## Abstract

Grapevine pathogens, such as *Plasmopara viticola*, a downy mildew disease-causing agent, pose a significant threat to grape and wine production worldwide. Molecular details are needed on how tolerant grapevine species recognize and mount a successful defense response against this pathogen. We have identified the important role of grapevine subtilase VviSBT4.19; however, without a reliable structural model, we have a limited understanding of protease activity modulation by *P. viticola*. Here, we built a consensus computational model of this protein and performed constant-pH MD simulations to assess its overall structural stability. We identified Ser419 as the key residue in autocatalytic processing and demonstrated that its S419N mutation produces a stable, active protease with a reduced autocatalytic activity. This work provides the first reliable structural model of VviSBT4.19, offering crucial insights into its function and regulation and opening new avenues for engineering enhanced disease resistance in grapevine cultivars.

## Graphical abstract

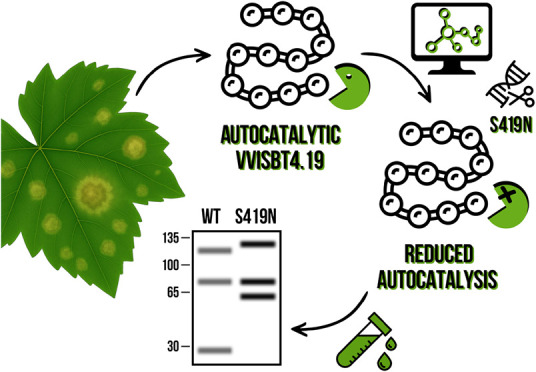

## Introduction

Grapevine (*Vitis vinifera*) cultivation is of many aspects of both cultural and economic interest. However, grapevine cultivation is highly susceptible to a wide range of pathogens, which can drastically reduce both crop yield and quality. Among the most economically damaging pathogens is downy mildew, caused by the oomycete *Plasmopara viticola*.^1^ This infection can drastically reduce crop yields, and, if left untreated, can result in complete production loss.^2,3^ The use of chemical methods, mainly fungicides, has been commonly employed to control such losses.^4^ However, a heavy dependence on these methods poses a significant risk to both the environment and human health. Excessive use of fungicides may lead to soil and water contamination, the loss of biodiversity, and the development of pesticide-resistant pathogen strains, all of which reduce the effectiveness of such treatments in the long run.^5,6^ Hence, it is urgent to develop new methods for sustainably managing this disease and ensuring grapevine production with reduced ecological impact in wine-producing regions.

Like other plants, grapevines have developed a multilayered immune system in response to pathogen attacks, employing various defense mechanisms that recognize and counter microbial invaders. The pattern-triggered immunity (PTI) and effector-triggered immunity (ETI) are two significant plant immunity pathways with major roles in plant defense against pathogen attacks.^7,8^

Proteomic studies have started to unveil the modulation of grapevine proteins during their interaction with *Plasmopara viticola*.^9–14^ Plant proteases have emerged as relevant players in plant immunity, in particular in grapevine immunity.^15–18^ Among such responses, proteases—particularly serine proteases—employ both regulatory and effector functions at critical steps of both PTI and ETI immune responses and thus have a direct impact on the ability of the plant to resist a pathogen infection.^19–23^ Plant subtilases, specifically, have been described as critical mediators of grapevine defense. Subtilases make up a large family of serine proteases that participate in the early detection of pathogen attacks and the activation of downstream defense responses. Figueiredo et al. have shown that this class of proteases is closely linked with jasmonic acid signaling, a hormone pathway vital for resistance against necrotrophic fungi and herbivores.^24^ In the following work, the same authors provided evidence that subtilases are not only upregulated following pathogen challenge but are also intricately involved in modulating the balance between defense responses and growth, an important consideration for sustainable breeding strategies.^11,23^ Their differential expression in response to pathogen aggressiveness is well documented. Distinct subtilase isoforms display varied expression patterns when challenged with different isolates of *P. viticola*, which underscores their role in early defense mechanisms.^14,25^ In particular, the grapevine subtilase VviSBT4.19 X1 demonstrates a marked increase in expression shortly after pathogen inoculation, suggesting its direct involvement in orchestrating an effective defense response.^22,26^

Although VviSBT4.19 has already been identified as playing a key role in grapevine immunity, a comprehensive structural and functional understanding of its role in pathogen resistance remains quite limited. This is primarily due to the absence of a reliable structural model, most likely resulting from autoproteolytic activity,^27^ which hinders in-depth analysis of how the protease interacts with its substrates and contributes to the pathogen defense process. Therefore, this work aims to fill this knowledge gap by building a computational model of VviSBT4.19, investigating its structural stability through molecular dynamics (MD) simulations, and devising mutants that lack catalytic activity and can be expressed and characterized experimentally. By providing a reliable structural model of VviSBT4.19, we help to investigate the molecular mechanism through which this protease executes its role in grapevine resistance against *P. viticola*. This may prove particularly useful in guiding future experimental studies focused on the peptide-binding affinity and bond specificity to engineer disease-resistant grapevine cultivars.

## Methods

### Structural Modeling

There is no experimentally determined structure for the VviSBT4.19 protein. Therefore, we resorted to different computational methods for structural prediction. We first generated a model using AlphaFold.^28^ However, because there is some uncertainty regarding the templates used during the alignment step, AlphaFold may incorporate sequences with high similarity but unrelated functions, potentially leading to nonoptimal predictions. To rule out this possibility, we also constructed a second structure using homology modeling. We identified experimental structures with high sequence similarity to VviSBT4.19 using the UniProt BLAST platform (https://www.uniprot.org/blast) with the UniProtKB database and the BLASTp algorithm. The search implemented the BLOSUM62 substitution matrix and was limited to 500 hits. Among the results, the structure with the highest sequence identity (PDB: 4YN3, cucumisin protease from melon fruit, 59% identity) was selected for homology modeling using MODELER software.^29^ The final model was chosen based on the fewest residues in disallowed regions of the Ramachandran plot. Considering that the full length of the protein contains an inhibitory domain (I9) that needs to be cleaved before activation of the protein, this domain, corresponding to the first 120 residues,^24^ was truncated from the protein.

### System Setup and MM/MD Settings

Simulations were performed using the GROMACS 5.1.5 package^30^ and the GROMOS 54A7 force field.^31^ The system was energy-minimized utilizing a two-step procedure of 10,000 integrator steps each, employing the steepest descent algorithm for both steps. The first of these steps was unconstrained, and the second one was performed with p-LINCS^32^ and SETTLE^33^ constraining algorithms applied on all bonds to the solute and solvent, respectively. The system was then initialized in a three-step procedure. This consisted of a 100 ps NVT MD step, with the v-rescale thermostat^34^ set for a reference temperature of 310 K, and a temperature coupling of 0.05 ps. Starting velocities were assigned from a Maxwell distribution representative of 310 K. This was followed by 100 ps of NPT MD with the Parrinello–Rahman isotropic barostat^35^ set for a reference pressure of 1 bar and pressure coupling of 1 ps. Both of these steps used an integration time of 1 fs. The long-range electrostatics were treated with the Particle Mesh Ewald (PME) method,^36^ with a Verlet cutoff scheme of 1.4 nm, a Fourier space grid of 0.12 nm, and an interpolation order of 4, and the neighbor list was updated every 10 steps. van der Waals interactions were truncated at 1.4 nm. The last step consisted of 100 ps of NPT MD with a temperature coupling of 0.1 ps, a pressure coupling of 2 ps, and an integration step of 2 fs, conditions adopted for all subsequent MD steps.

### Poisson–Boltzmann/Monte Carlo and CpHMD Settings

Constant-pH molecular dynamics (CpHMD) simulations are beneficial since the starting structure of the VviSBT4.19 protein was generated using a homology modeling procedure. There is additional uncertainty regarding the positioning of the side chains and their local environments, which, in turn, affects their most abundant protonation states. Without a good initial guess, we resorted to a method that updates protonation states on the fly and maintains the protein’s structural stability.

Our implementation of the CpHMD method^37–43^ couples conformational sampling from MD simulations with protonation-state sampling from Poisson–Boltzmann/Monte Carlo (PB/MC) calculations. In this method, a CpHMD cycle begins with a PB/MC step, where we calculate the free energies of each protonation (tautomer) state for our titrating groups using the DelPhi V5.1 program.^44^ We use a dielectric constant of 2 ^45,46^ to address the lack of polarization effects in a fixed charge model such as the one used, while the solvent is treated implicitly and with a high dielectric constant of 80.^45,47,48^ The molecular surface of the solute was defined by using a probe with a radius of 1.4 Å. The populations of each tautomer within each protonation state are sampled using PETIT v1.6.1,^49^ which employs an MC scheme based on free energy terms calculated in the previous PB step. A total of 10^5^ MC cycles were performed for each conformation, with the protonations of the last cycle selected as the new protonation states. The next step in the CpHMD cycle is a short solvent-relaxation MD step of 0.2 ps, with the solute frozen, to allow solvent molecules to adjust to the new protonation states. The cycle is then completed with a 20 ps production MD segment that samples the system’s conformational space with the new solute protonation states. This procedure is then iterated.

Since long-range electrostatics are treated with PME,^36^ simulations require charge neutrality. An initial estimate of the number of counterions was obtained from the PypKa tool,^50,51^ which provided a protein total titration curve mapping the protein charge at the target pH. We submitted our protein structure model to the PypKa web server^52^ and obtained a total charge of −10 at pH 7.0. We solvated our system, containing the protein structure obtained from homology modeling in a rhombic dodecahedral box, with 24716 water molecules using the SPC water model, applying periodic boundary conditions. From the number of water molecules and the volume of a water molecule (≈3 × 10^–26^ dm^3^), we calculated the total ions (134) to achieve a cell-like ionic strength of 150 mM. This leads to 72 Na^+^ (62 + 10 to neutralize the system) and 62 Cl^–^ ions added to the system. It is worth noting that this approach typically involves iterative steps of performing short CpHMD segments to evaluate and adjust the number of counterions, thereby maintaining the system charge near neutrality. The convergence of this procedure depends on how representative the structure provided to PypKa is of the protein in solution.

All aspartic acid, glutamic acid, and histidine residues were titrated, while all remaining titratable residues had their protonations fixed to the most probable state at the simulated pH (all lysine and tyrosine residues were protonated). We performed 200 ns of production simulations at pH 7.0, with 5 replicates. Additionally, a variant of the protein with a Ser-to-Asn mutation at position 419 was built *in silico* and prepared for CpHMD simulations. All settings for these simulations were the same as those previously described.

### Computational Analyses

All structural properties were calculated using GROMACS^30^ or in-house tools. All structures were visualized and rendered using PyMOL^53^ and plotted with Gnuplot.^54^ Error values were calculated by using the standard error of the mean.

#### Cross-RMSD

Since our focus is on the catalytic triad of this protein, it is important to evaluate whether the S419N mutation impacts the protein’s fold and the catalytic pocket. Hence, we identified and selected the protein’s core residues by calculating the root-mean-square fluctuation (RMSF) of each residue over time (Figure S1 of the Supporting Information). A cutoff of <0.15 nm was used to select residues with lower fluctuations, thereby identifying the protein’s core. Then, we calculated the root-mean-square deviation (RMSD) of the cross-combinations between the final frame of each replicate of the *wt* and mutant proteins for the C_α_ atoms of the previously identified core residues.

#### Energy Landscapes of Catalytic Triad Activation

The conditional free energy landscapes were calculated from a probability density function over a 2D space using His–Ser and His–Asp distances as structural coordinates. The probability density functions were estimated using a Gaussian kernel estimator with a grid spacing of 0.05 Å^3^.^55^ The conditional energy (*E*_(r)_) surfaces were computed with the following equation

1 $$E_{\left(r\right)}=-\mathrm{RT}\frac{\ln P_{\left(r\right)}}{P_{\max}}$$

where *R* and *T* are the ideal gas constant and temperature, respectively, while *P*_(r)_ and *P*_max_ are the probability density function and its maximum.

### Experimental Details

#### Cloning and Mutation Procedures of VviSBT4.19

The Open Reading Frame (ORF) of VviSBT4.19 was amplified from a cDNA sample of *V. vinifera* cv. “Regent” inoculated with *P. viticola*. Gene amplification was performed by polymerase chain reaction (PCR) using a 50 μL reaction mix containing 1 μL of cDNA, 0.5 μM of each specific primer, 0.25 mM each dNTP, 0.02 U/μL of Phusion High-Fidelity DNA Polymerase (Thermo Scientific, Waltham, Massachusetts, USA), and 1X Phusion HF Buffer. Thermal cycling began with a denaturation step at 98 °C for 30 s, followed by 35 cycles of denaturation at 98 °C for 10 s, annealing at 60 °C for 30 s, extension at 72 °C for 90 s, and final extension at 72 °C for 10 min. After amplification, the ORF was cloned into pJET1.2/blunt vector using a CloneJET PCR Cloning Kit (Thermo Scientific, USA), according to the manufacturer’s instructions, resulting in pJET1.2.VviSBT4.19. Using the Gateway Cloning system (Gateway Technology), VviSBT4.19 ORF was cloned into the pGWB405 vector (Addgene, USA), resulting in construct pGWB405.VviSBT4.19. Positive clones were identified by colony PCR and sequenced (STABVIDA, Caparica, Portugal). Site-directed mutagenesis was performed using the Q5 Site-Directed Mutagenesis Kit (New England Biolabs), following the manufacturer’s instructions. A 60 min KLD reaction was carried out, followed by an additional inactivation step at 80 °C for 10 min. This resulted in the mutated construct, pGWB405.VviSBT4.19.mut. *Escherichia coli* DH5α cells were transformed with all of the vectors mentioned, while *A. tumefaciens* AGL1 was transformed with pGWB405.VviSBT4.19, pGWB405.VviSBT4.19.mut, and the empty pGWB405 vector. The primers used for cDNA synthesis, cloning, and site-directed mutagenesis are listed in Table S1 of the Supporting Information.

#### Plant Material

*Nicotiana benthamiana* seeds were soaked in distilled water for 1 h in a Petri dish and then transferred to pots containing a soil-to-vermiculite mixture (3:1). Pots were placed in a humid chamber, in a 25 °C greenhouse with a 16h/8h light/dark cycle period and 60% of humidity for 2 weeks, until germination was observed. Subsequently, each seedling was transplanted into a new pot with the same soil–vermiculite mix and grown under 25 °C with the same light/dark periods and 60% of humidity.

#### Agroinfiltration of *N. benthamiana*

Agrobacterium-mediated transient expression in *N. benthamiana* plants was used for the production of recombinant protein. *A. tumefaciens* AGL1 wild type (*wt*), pGWB405 vectors, and the P19 silencing suppressor were grown in LB liquid media supplemented with the specific antibiotics overnight at 28 °C and 200 rpm. Cells were harvested by centrifugation (5000 rpm for 10 min), resuspended in infiltration medium (0.01 M MES at pH 5.6; 0.01 M MgCl_2_ and 200 μM acetosyringone) with an OD600 nm of 0.5, and incubated for 60 min at 28 °C and 150 rpm. *A. tumefaciens* AGL1 cultures were mixed with the Agrobacterium strain carrying the P19 silencing suppressor in a 1:1 ratio immediately before infiltration. *N. benthamiana* leaves, from 4 to 6-week-old plants, were agroinfiltrated with this mixture. Plants were incubated under the same conditions as those described above. Four days after infiltration, leaves were collected by freezing in liquid nitrogen and stored at −80 °C. Three *N. benthamiana* plants were used per condition (*wt*, pGWB405 vectors, and P19). The agroinfiltration procedure was repeated in four independent experiments.

#### Fluorescence Microscopy

Four days after infiltration, leaves were collected for confocal microscopy studies. Confocal microscopy was performed by using a Leica TCS SP5 system equipped with a 488 nm laser line for GFP excitation. Images were acquired with Leica TCS SP5 software and processed using Fiji (Fiji Is Just ImageJ).

#### Protein Quantification

Protein abundance was quantified from fluorescence images acquired as previously described, using Fiji software with the macro provided in the Supporting Information.

#### Western Blot Analysis

*N. benthamiana* leaves transformed with pGWB405 vectors were ground in liquid nitrogen, and proteins were extracted with the extraction buffer (100 mM carbonate–bicarbonate buffer pH 9.5, 50 mM NaCl, 0.1% tween20, 5 mM DTT, and protease inhibitors cocktail composed of 0.05 mM E-64, 0.05 mM Pestatin A, 50 μM Cystatin, 5 mM Phenanthroline, 5 mM EDTA). Protein extracts were centrifuged for 14,000*g* for 10 min at 4 °C. Sample buffer (0.08 M Tris–HCl pH 6.8, 2% SDS, 5% β-mercaptoethanol, 10% glycerol, 0.001% bromophenol blue) was added in a 1:4 ratio to the protein extract supernatant. Protein samples were then boiled for 5 min at 95 °C. Samples were resolved by SDS-PAGE and transferred to a nitrocellulose membrane. GPF fusion proteins were detected with α GFP antibody (3H9, Proteintech, Germany) diluted 1:1000 in PBS-T and incubated for 1 h at room temperature with gentle shaking. The membrane was washed three times with PBS-T for 5 min and incubated with an HRP-conjugated Goat Anti-Rat IgG­(H + L) (SA00001–15, Proteintech, Germany) for 2 h at room temperature with gentle shaking. Signal was detected by chemiluminescence using Amersham ECL Prime reagent (GE Healthcare Life Sciences, U.K.) in an Amersham Imager 680 (GE Healthcare, USA).

#### Protein Isolation and Activity Assessment

*Wt* and mutant VviSBT4.19 proteins were isolated through NanoTag’s GFP Selector Resin (N0310, Proteintech, Germany), following the manufacturer’s batch protocol. Briefly, native protein extracts were prepared from *N. benthamiana* leaves and clarified by centrifugation at 14,000*g* for 10 min at 4 °C. Lysates were incubated with 10 μL of pre-equilibrated GFP Selector Resin (in protein extraction buffer) for 1 h at 4 °C under head-overtail rotation. Beads were subsequently washed twice with protein extraction buffer and then three times with PBS. Bound proteins were eluted by resuspending beads in 50 μL of 2× SDS sample buffer, heating at 95 °C for 5 min, and centrifuging at 3000*g* for 1 min. The supernatant was collected, and 48 μL was labeled with 1 μL of 2 μM ActivX TAMRA-FP Serine Hydrolase Probe (88318, Thermo–Fischer Scientific, USA) and 1 μL of 250 mM DTT. Samples were labeled for 5 h at room temperature in a tube rotator. Then, 200 μL of ice-cold acetone was added to the samples, which were then vortexed. Samples were centrifuged at 14,000*g* for 2 min, and then the protein pellet was resuspended in sample buffer. Samples were resolved in an SDS-PAGE gel, and bands were imaged in an Amersham Imager 680 (Thermo-Fischer Scientific, USA).

## Results and Discussion

### Structural Model of the VviSBT4.19 Subtilase Protein

The initial goal of this work was to obtain an adequate computational model for the VviSBT4.19 structure. The strong autocatalytic activity has limited all efforts to purify and further study the structure of this protein. However, due to its key role in the grapevine defense mechanism against pathogens, it is crucial to overcome this hurdle and gain insight into the molecular details of this enzyme’s mode of action. We built two computational models: one using AlphaFold and another following a classical homology modeling protocol. This double approach also provides important validation of our computational protocol. The two structures are in very good agreement (Figure S2 of Supporting Information) with only a few differences located in the loop regions of the protein. We opted to use the homology model (Figure 1) in the CpHMD simulations, as its bias toward a specific experimental structure (cucumisin protease from melon fruit) with high homology may provide better packing of the residues’ side chains.

**Figure 1:**
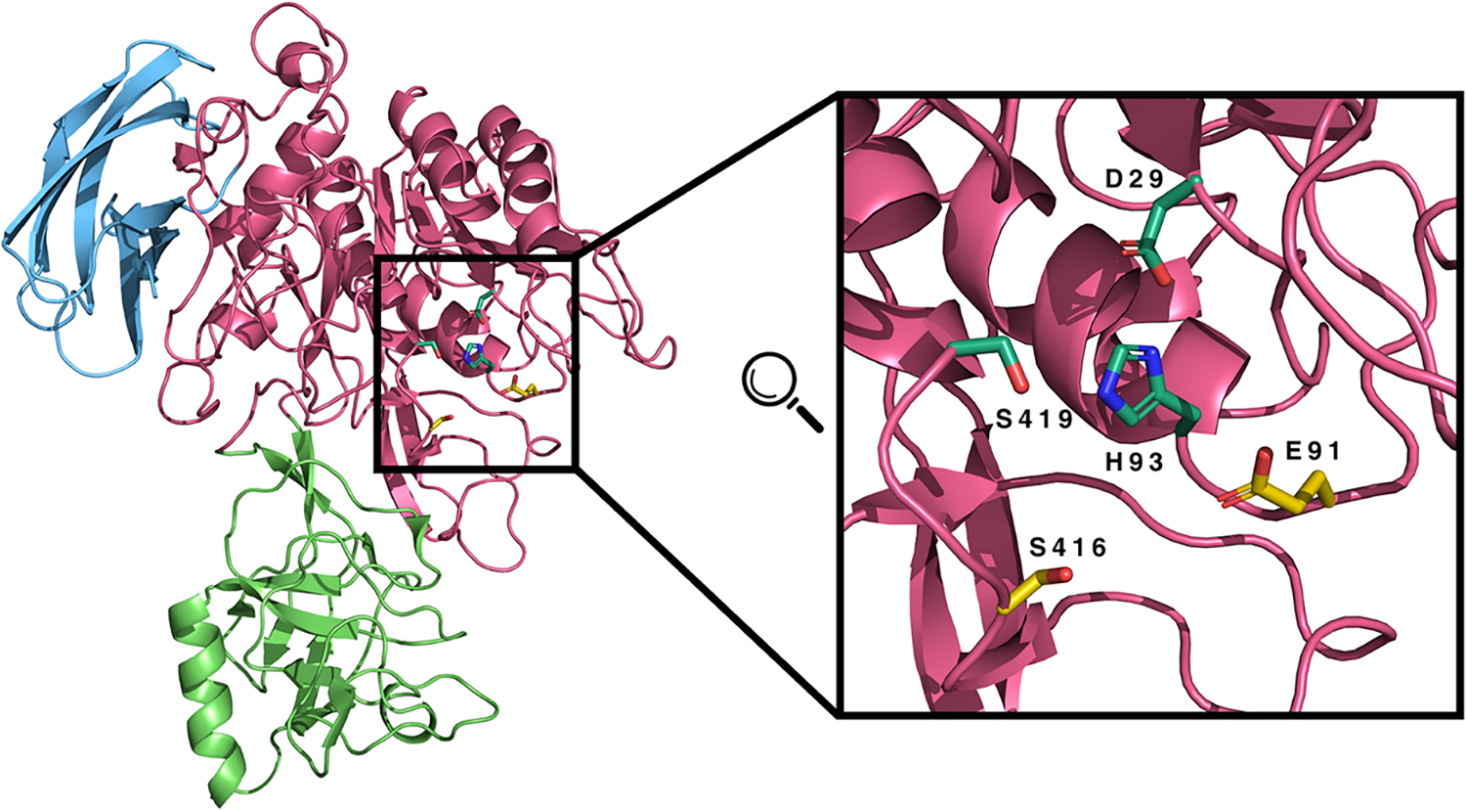
Cartoon representation of the Modeler-generated structure, colored in pink. Only residues 121 to 746 are displayed. The I9 domain (1–120) was excluded since it is unstructured and misplaced within an internal cavity in the best-obtained model. The PA domain (341–478) and the FN-II domain (649–746) are highlighted in green and blue, respectively. On the right, a zoomed-in view of the catalytic pocket is depicted, with key residues represented as sticks. The putative catalytic triad (D29, H93, and S419) is highlighted in greenish-blue, while nearby residues (E91 and S416), which may form an alternative triad with H93, are shown in yellow.

We started by evaluating the equilibration and convergence of our CpHMD simulations and also assessed the quality of the structural model proposed. We used standard structural metrics, including the RMSD of the protein’s core, radius of gyration (*R*_g_), secondary structure, and the protein’s total charge (Figure S3 of Supporting Information). The structural properties showed overall stability of the model, characterized by low RMSD (<0.3 nm), and regular radius of gyration (∼2.6 nm) and secondary structure contents (helicity and β-structure). The small differences observed in some replicates are negligible, typically due to variations in the packing of loop regions, and do not significantly affect the overall convergence of our simulations. Regarding the total protein charge, we observe stable fluctuations around −10 (Figure S3D of the Supporting Information). This also validates our initial guess of using an excess of 10 cations to ensure that our system charge fluctuates around neutrality. All properties equilibrate within the initial 50 ns of our simulations, and this segment was discarded for averaging purposes.

When our model was aligned with the template, we identified residues Ser419, His93, and Asp29 to constitute a catalytic triad for a typical serine-protease-like protein (Figure 1). However, a visual inspection of the active site also found another Ser/Acid pair (Ser416/Glu91) near the catalytic histidine, which could constitute an alternative catalytic triad (Figure 1). To investigate this possibility, we calculated the distances between the elements of these two triads and evaluated their suitability (proximity) to perform catalysis (Figures 2 andS4 of the Supporting Information). The energy landscapes of the distances to His93 show that the Ser419/Asp29 pair samples mostly short distances (both within ∼7 Å), which are more suitable for catalysis (Figure 2A). A total of 79% of the conformations fulfill this criterion. On the other hand, during our simulation, the Ser416/Glu91 pair did not come together or align at distances compatible with catalysis (Figure 2B). The evidence favoring the Ser419/Asp29 pair is strong; however, it is worth noting that this analysis was performed with the protein in its apo form, and these energy landscapes may change once a substrate enters the catalytic pocket. Therefore, these findings still require confirmation from experimental data.

**Figure 2:**
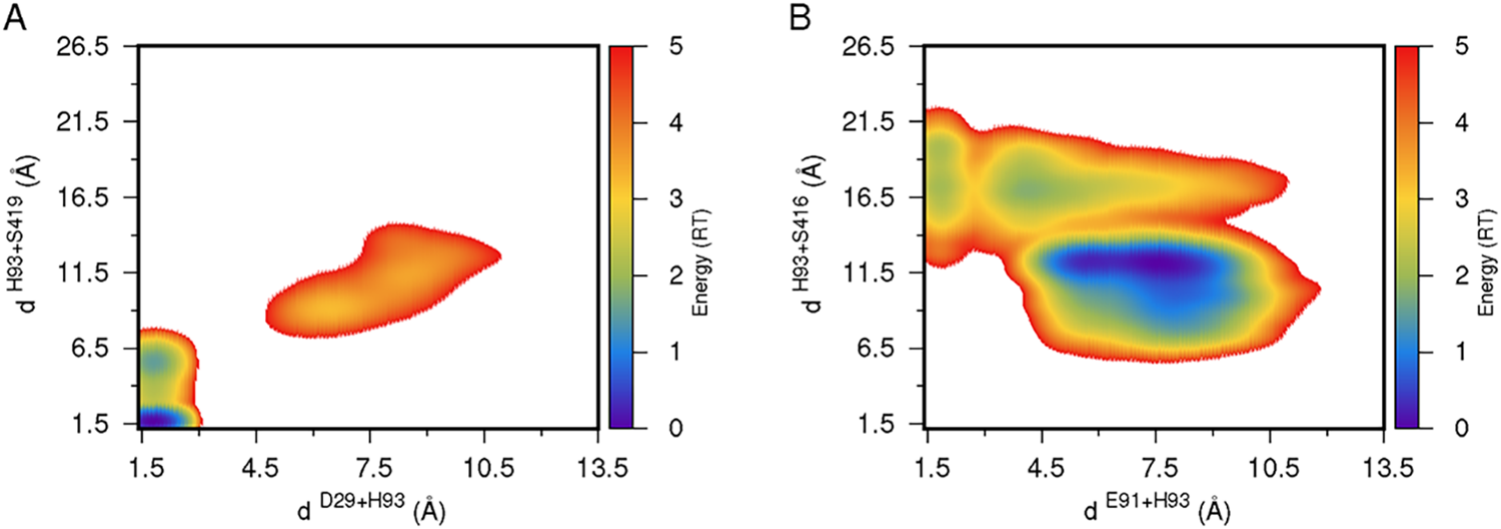
Energy landscapes of the distances between the Asp29-His93 and His93-Ser419 residue pairs (A) and between the Glu91-His93 and His93-Ser416 pairs (B). The energy scale is shown as a color palette on the right.

Our best model identifies Ser419 as the key catalytic residue and a potential target for site-directed mutagenesis to inhibit catalysis. From an experimental standpoint, a mutation that turns off (or weakens) the autocatalytic activity (self-degradation) is essential for producing the protein and performing any structure-dependent experimental analysis. We proposed the S419N mutation for this effect and performed CpHMD simulations with this system.

All structural properties of the S419N mutant remain very stable (Figure S5 of the Supporting Information), similar to the *wt* system (Figure S3 of the Supporting Information). Regarding the system’s charge, we observe stable fluctuations around the same value as in the *wt* (−10). This confirms that the mutation does not significantly impact the overall protein stability and electrostatics. In the catalytic pocket, we have already shown that the triad comprising Glu91, His93, and Ser416 is not aligned for catalysis. However, with the mutation, the protein may undergo structural rearrangements to activate the second triad that remains present. Hence, we calculated these distances to His93 (Figure S6 of Supporting Information) and plotted the resulting energy landscape (Figure 3A). This energy landscape shows that most conformations have distances that consistently exceed reasonable values for catalysis, as evidenced by the large blue cluster. Only the Glu91–His93 pair transiently populates adequate distances (below 3 Å) without approaching Ser416, hence without activation. It is worth noting that the observable clusters follow a similar pattern to the ones observed for this triad in the *wt* system (Figure 2B). This further indicates that the S419N mutation has little to no structural impact on the protein’s catalytic pocket. To further evaluate this impact, we calculated the cross-RMSD values of the protein core between *wt* and S419N (Figure 3B). The results show that the average structural dissimilarity (RMSD) between *wt* and the mutant is the same across all of the conformational ensembles. This confirms that the protein is populating the same conformational space, even after the S419N mutation. Overall, our results indicate that this mutation should provide an excellent structural model for the VviSBT4.19 serine protease, in which most autocatalytic activity is suppressed, allowing the protein to be experimentally tested.

**Figure 3:**
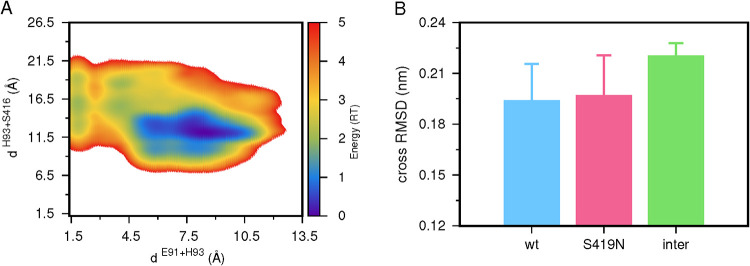
Energy landscape of the distances between the Glu91–His93 and His93–Ser416 pairs for the S419N variant (A) and a cross-RMSD analysis between the serine protease *wt* and S419N mutant (B). The energy landscape scale is shown as a color palette. The cross-RMSD analyses were performed between the five replicates within the *wt* (light blue) and S419N (magenta) or across (inter) both systems (green).

### Functional Validation of the S419N Mutation

To experimentally validate the predicted catalytic role of Ser419, we generated a site-directed S419N mutant designed to disrupt the catalytic triad without compromising the protease’s overall structural integrity, as confirmed by CpHMD simulations. Both *wt* and mutant forms of VviSBT4.19 were transiently expressed as C-terminal GFP fusion proteins in *N. benthamiana* leaves. *N. benthamiana* was chosen since it is the standard heterologous expression system for transient assays in plants, offering high transformation efficiency, fast protein accumulation, and reliable expression of secreted and apoplastic proteases.^56^

Confocal fluorescence microscopy revealed a markedly stronger GFP signal in leaves expressing the S419N mutant compared with the *wt* construct (Figure 4), consistent with reduced proteolytic degradation. Quantitative fluorescence measurements (Figure 4C) supported this observation, which indicates that accumulation of the S419N mutant is significantly higher than that of the VviSBT4.19. In addition, the fluorescence signal of the mutated protein appeared to accumulate predominantly at the cell periphery, suggesting cell wall, plasma membrane, or apoplastic localization, as well as in the nuclei. Although compartment-specific markers were not used, this distribution may reflect altered trafficking or retention of the stabilized protein variant. Several subtilases have already been reported to be targeted to the cell wall, where they play roles in regulating cell wall properties and extracellular signaling. This localization is crucial for processes such as growth, development, and response to environmental stimuli.^27^ Subtilases such as *Arabidopsis* SBT3.3 and SBT3.5 accumulate in the cell wall, where they activate pectin methylesterase (PME) activity, thereby contributing to plant immunity. This extracellular localization is essential for their role in modulating cell wall integrity and defense responses against pathogens.^57^

**Figure 4:**
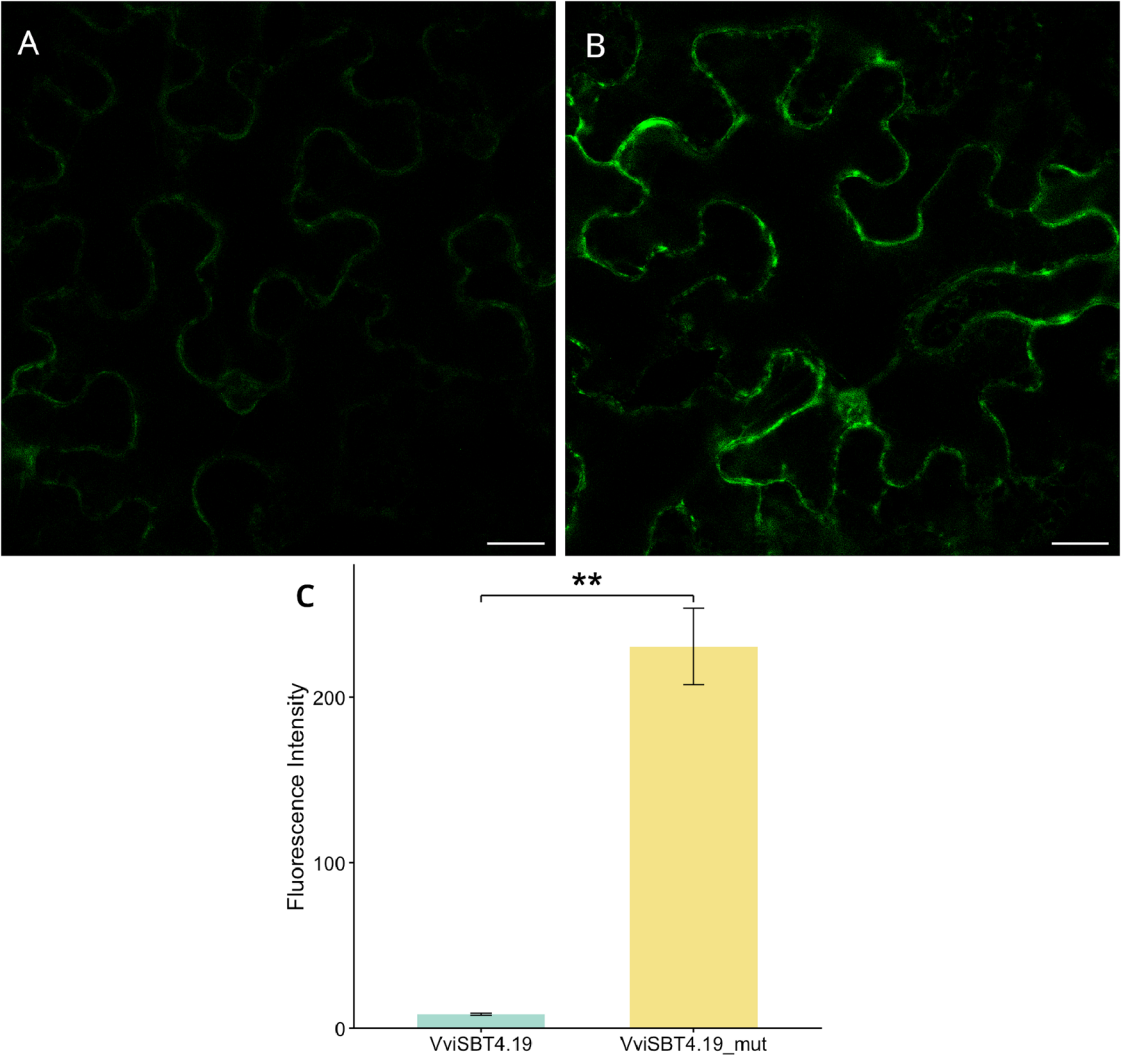
Confocal microscopy imaging of *N. benthamiana* leaf cells expressing VviSBT4.19:GFP (A) and VviSBT4.19.mut:GFP (B). Scale bar: 20 μm. Whole-image fluorescence in VviSBT4.19 and its S419N mutation (VviSBT4.19_mut) plants (C). Statistical significance between groups was assessed using Mann–Whitney U test; “**” indicates *p* < 0.01.

To further investigate the differences in protein processing, a Western blot analysis with anti-GFP antibodies was performed (Figure 5). The *wt* VviSBT4.19 protein produced three bands: one corresponding to free GFP (27 kDa) and two at ∼50 and ∼110 kDa. The first two bands are indicative of proteolytic cleavage. The mutated protein also showed three bands with different molecular weights: a prominent intact fusion protein at ∼110 kDa, along with bands at ∼70 and ∼50 kDa. The full-length fusion protein is present in both *wt* and mutated proteins; however, it appears to accumulate at higher levels. This may suggest that the S419N mutation alters some of the autocatalytic activity, leading to higher accumulation of the more stable mutant protein. To directly assess protease activity, we performed an activity-based protein profiling (ABPP) assay using the probe FP-TAMRA, which covalently labels active serine hydrolases. The purified *wt* and S419N proteins were incubated with FP-TAMRA and resolved by SDS-PAGE, followed by fluorescence detection using an Amersham Imager. The *wt* enzyme displayed three fluorescent bands: a major band around 110 kDa and two additional bands at ∼80 and ∼45 kDa. In contrast, the mutant protein produced four fluorescent bands: a higher band at ∼110 kDa (slightly above the *wt* major band) and three additional bands at ∼63, ∼50, and ∼45 kDa. The altered banding pattern, particularly the upward shift of the highest molecular weight band and the appearance of distinct lower bands in the mutant, suggests changes in processing or labeling efficiency due to loss of catalytic activity.

**Figure 5:**
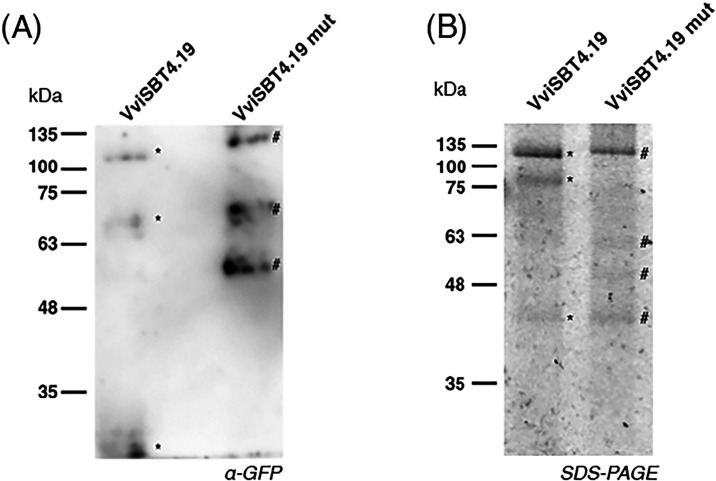
Western blot analysis (A) and activity-based protein profiling (B) of *wt* and mutant VviSBT4.19. * indicates *wt* and # indicates mutant VviSBT4.19 bands.

These observations collectively indicate that S419 corresponds to the catalytic serine of the conserved Asp-His-Ser catalytic triad in VviSBT4.19. In the well-characterized tomato subtilase SlSBT3, mutation of the analogous catalytic serine (S538) to alanine or cysteine completely abolishes autocatalytic prodomain removal, prevents secretion, and eliminates proteolytic activity.^58,59^ Our S419N mutant displays a qualitatively similar but quantitatively milder phenotype, consistent with the more conservative nature of the Ser → Asn substitution. The accumulation of the ∼110 kDa full-length precursor, the absence of free GFP release, and the appearance of the novel ∼70 kDa intermediate all point to impaired autocatalytic processing, which is a hallmark of catalytic serine dysfunction in plant subtilases.^58,60^ The FP-TAMRA labeling in the S419N mutant confirms that serine hydrolase activity was almost abolished, with only residual activity and altered specificity, as evidenced by a shifted molecular weight distribution and distinct processing intermediates. This strong loss-of-function phenotype is particularly informative, as it reveals normally transient processing intermediates and demonstrates that an impairment of the catalytic serine profoundly affects autocatalytic maturation, protein stability, and cleavage site selection in plant subtilases.^58,60^

The structural model obtained in this work establishes a robust framework for studying pathogen-mediated modulation of protease activity during infection, thereby also advancing our mechanistic understanding of host–pathogen interactions. Indeed, the structural model will contribute to identifying putative substrates through computational docking and molecular dynamics simulations, enabling prediction of cleavage sites and substrate specificities that define VviSBT4.19’s role in grapevine defense responses. By integrating structural information with substrate profiling approaches, future studies can elucidate the specific proteolytic cascades and signaling pathways through which VviSBT4.19 mediates resistance to biotic stress. Such insights will not only clarify the molecular mechanisms underlying subtilase function in plant immunity but also inform the rational design of strategies to enhance pathogen resistance in grapevines and other crops.

## Supplementary Material







## Notes

List of DNA primer sequences used in this work; structural models generated with AlphaFold and Modeler; time series of RMSD, *R*_g_, secondary structure content, total charge, and distances between catalytic residues for both the *wt* and the S419N mutation (PDF)
Topologies, system configurations, and parameters (ZIP)
Fiji macro and the resulting Excel spreadsheet with the protein quantification (ZIP)

## References

[1] Koledenkova K., Esmaeel Q., Jacquard C., Nowak J., Clément C., Ait Barka E. (2022). *Plasmopara viticola* the
causal agent of downy mildew of grapevine: from its taxonomy to disease
management. Front. Microbiol..

[2] Gessler C., Pertot I., Perazzolli M. (2011). *Plasmopara viticola*: a review of knowledge on downy
mildew of grapevine and effective
disease management. Phytopathol. Mediterr..

[3] Savary S., Willocquet L., Pethybridge S. J., Esker P., McRoberts N., Nelson A. (2019). The global burden of pathogens and pests on major food
crops. Nat. Ecol. Evol..

[4] Dagostin S., Schärer H.-J., Pertot I., Tamm L. (2011). Are there alternatives
to copper for controlling grapevine downy mildew in organic viticulture?. Crop Prot..

[5] Lamichhane J. R., Dachbrodt-Saaydeh S., Kudsk P., Messéan A. (2016). Toward a reduced
reliance on conventional pesticides in European agriculture. Plant Dis..

[6] Komárek M., Čadková E., Chrastnỳ V., Bordas F., Bollinger J.-C. (2010). Contamination of vineyard soils with
fungicides: a review of environmental and toxicological aspects. Environ. Int..

[7] Ngou B. P. M., Ahn H.-K., Ding P., Jones J. D. (2021). Mutual
potentiation
of plant immunity by cell-surface and intracellular receptors. Nature.

[8] Wu S., Shan L., He P. (2014). Microbial signature-triggered plant
defense responses and early signaling mechanisms. Plant Sci..

[9] Nascimento-Gavioli M. C. A., Agapito-Tenfen S. Z., Nodari R. O., Welter L. J., Mora F. D. S., Saifert L., da Silva A. L., Guerra M. P. (2017). Proteome
of *Plasmopara viticola*-infected *Vitis vinifera* provides insights into grapevine Rpv1/Rpv3
pyramided resistance to downy mildew. J. Proteom..

[10] Figueiredo A., Martins J., Sebastiana M., Guerreiro A., Silva A., Matos A. R., Monteiro F., Pais M. S., Roepstorff P., Coelho A. V. (2017). Specific adjustments
in grapevine
leaf proteome discriminating resistant and susceptible grapevine genotypes
to *Plasmopara viticola*. J. Proteom..

[11] B
Santos R., Nascimento R., V Coelho A., Figueiredo A. (2020). Grapevine–downy
mildew rendezvous: Proteome analysis of the first hours of an incompatible
interaction. Plants.

[12] Liu G.-T., Wang B.-B., Lecourieux D., Li M.-J., Liu M.-B., Liu R.-Q., Shang B.-X., Yin X., Wang L.-J., Lecourieux F., Xu Y. (2021). Proteomic analysis of early-stage
incompatible and compatible interactions between grapevine and *P. viticola*. Hortic. Res..

[13] Figueiredo J., Santos R. B., Guerra-Guimarães L., Leclercq C. C., Renaut J., Malhó R., Figueiredo A. (2022). An in-planta
comparative study of *Plasmopara viticola* proteome reveals different infection strategies towards susceptible
and Rpv3-mediated resistance hosts. Sci. Rep..

[14] Figueiredo J., Santos R. B., Guerra-Guimarães L., Leclercq C. C., Renaut J., Sousa L., Figueiredo A. (2023). Deep into
the apoplast: Grapevine and *Plasmopara viticola* proteomes reveal the secret beneath host and pathogen communication
at 6 h after contact. Phytopathology.

[15] Santos R. B., Figueiredo A. (2021). Two sides
of the same story in grapevine–pathogen
interactions. J. Exp. Bot..

[16] Figueiredo L., Santos R. B., Figueiredo A. (2021). Defense and
offense strategies: the
role of aspartic proteases in plant–pathogen interactions. Biology.

[17] Figueiredo L., Santos R. B., Figueiredo A. (2022). The grapevine aspartic protease gene
family: characterization and expression modulation in response to *Plasmopara viticola*. J. Plant
Res..

[18] Paiva-Silva C., Proença Pereira J., Marcolino F., Figueiredo A., Santos R. B. (2025). Silenced cutters: mechanisms and
effects of protease inhibition in plant-pathogen interactions. J. Exp. Bot..

[19] Misas-Villamil J. C., van der Hoorn R. A., Doehlemann G. (2016). Papain-like cysteine proteases as
hubs in plant immunity. New Phytol..

[20] Thomas E. L., Van der Hoorn R. A. (2018). Ten prominent
host proteases in plant-pathogen interactions. Int. J. Mol. Sci..

[21] Figaj D., Ambroziak P., Przepiora T., Skorko-Glonek J. (2019). The role of
proteases in the virulence of plant pathogenic bacteria. Int. J. Mol. Sci..

[22] Figueiredo A., Monteiro F., Sebastiana M. (2014). Subtilisin-like proteases in plant–pathogen
recognition and immune priming: a perspective. Front. Plant Sci..

[23] Figueiredo J., Silva M. S., Figueiredo A. (2018). Subtilisin-like
proteases in plant
defence: the past, the present and beyond. Mol.
Plant Pathol..

[24] Figueiredo J., Costa G. J., Maia M., Paulo O. S., Malhó R., Sousa Silva M., Figueiredo A. (2016). Revisiting *Vitis vinifera* subtilase gene family: a possible role in grapevine resistance against *Plasmopara viticola*. Front.
Plant Sci..

[25] Gouveia C., Santos R. B., Zukic S., Manthey T., Malhó R., Figueiredo A., Buchholz G. (2024). Novel *Plasmopara viticola* isolate surpasses grapevine Rpv3. 1 and Rpv3. 2 resistance but not
Rpv12. J. Plant Pathol..

[26] Figueiredo J., Cunha J., Eiras-Dias J., Silva M. S., Figueiredo A. (2020). Pathogen-related
specificity of subtilase VVISBT4. 19 X1 in the *Vitis
vinifera* defence response. Cienc.
Tec. Vitivinic..

[27] Schaller A., Stintzi A., Rivas S., Serrano I., Chichkova N. V., Vartapetian A. B., Martínez D., Guiamét J. J., Sueldo D. J., van Der
Hoorn R. A. (2018). From structure to function–a
family portrait of plant subtilases. New Phytol..

[28] Jumper J., Evans R., Pritzel A., Green T., Figurnov M., Ronneberger O., Tunyasuvunakool K., Bates R., Žídek A., Potapenko A. (2021). Highly accurate protein structure prediction
with AlphaFold. Nature.

[29] Šali A., Blundell T. L. (1993). Comparative protein
modelling by satisfaction of spatial
restraints. J. Mol. Biol..

[30] Abraham M. J., Murtola T., Schulz R., Páll S., Smith J. C., Hess B., Lindahl E. (2015). GROMACS: High
performance
molecular simulations through multi-level parallelism from laptops
to supercomputers. SoftwareX.

[31] Schmid N., Eichenberger A. P., Choutko A., Riniker S., Winger M., Mark A. E., Van Gunsteren W. F. (2011). Definition and testing of the GROMOS
force-field versions 54A7 and 54B7. Eur. Biophys.
J..

[32] Hess B. (2008). P-LINCS: A
Parallel Linear Constraint Solver for Molecular Simulation. J. Chem. Theory Comput..

[33] Miyamoto S., Kollman P. A. (1992). SETTLE: An analytical version of the SHAKE and RATTLE
algorithm for rigid water models. J. Comput.
Chem..

[34] Bussi G., Donadio D., Parrinello M. (2007). Canonical sampling through velocity
rescaling. J. Chem. Phys..

[35] Parrinello M., Rahman A. (1981). Polymorphic transitions in single crystals: A new molecular
dynamics method. J. Appl. Phys..

[36] Darden T., York D., Pedersen L. (1993). Particle mesh
Ewald: An Nlog­(N) method
for Ewald sums in large systems. J. Chem. Phys..

[37] Santos H. A. F., Vila-Viçosa D., Teixeira V. H., Baptista A. M., Machuqueiro M. (2015). Constant-pH
MD simulations of DMPA/DMPC lipid bilayers. J. Chem. Theory Comput..

[38] Teixeira V. H., Vila-Viçosa D., Reis P. B. P. S., Machuqueiro M. (2016). *pK*
_
*a*
_ Values
of Titrable Amino Acids at the
Water/Membrane Interface. J. Chem. Theory Comput..

[39] Reis P. B. P. S., Vila-Viçosa D., Campos S. R., Baptista A. M., Machuqueiro M. (2018). Role of counterions
in constant-pH molecular dynamics
simulations of PAMAM dendrimers. ACS Omega.

[40] Silva T. F. D., Vila-Viçosa D., Reis P. B., Victor B. L., Diem M., Oostenbrink C., Machuqueiro M. (2018). The impact
of using single atomistic long-range cutoff schemes with the GROMOS
54A7 force field. J. Chem. Theory Comput..

[41] Vila-Viçosa D., Reis P. B. P. S., Baptista A. M., Oostenbrink C., Machuqueiro M. (2019). A pH Replica
Exchange Scheme in the Stochastic Titration
Constant-pH MD Method. J. Chem. Theory Comput..

[42] Oliveira N. F. B., Machuqueiro M. (2022). Novel US-CpHMD
Protocol to Study the Protonation-Dependent
Mechanism of the ATP/ADP Carrier. J. Chem. Inf.
Model..

[43] Sequeira J. G. N., Rodrigues F. E., Silva T. G., Reis P. B., Machuqueiro M. (2022). Extending
the Stochastic Titration CpHMD to CHARMM36m. J. Phys. Chem. B.

[44] Rocchia W., Alexov E., Honig B. (2001). Extending the applicability
of the
nonlinear Poisson-Boltzmann equation: Multiple dielectric constants
and multivalent ions. J. Phys. Chem. B.

[45] Baptista A. M., Teixeira V. H., Soares C. M. (2002). Constant-pH
molecular dynamics using
stochastic titration. J. Chem. Phys..

[46] Henriques J., Costa P. J., Calhorda M. J., Machuqueiro M. (2013). Charge Parametrization
of the DvH-*c*
_3_ Heme Group: Validation Using
Constant-(pH, *E*) Molecular Dynamics Simulations. J. Phys. Chem. B.

[47] Machuqueiro M., Baptista A. M. (2006). Constant-pH Molecular Dynamics with Ionic Strength
Effects: Protonation–Conformation Coupling in Decalysine. J. Phys. Chem. B.

[48] Machuqueiro M., Baptista A. M. (2011). Is the prediction of *pK*
_
*a*
_ values by constant-pH molecular dynamics being hindered
by inherited problems?. Proteins Struct. Funct.
Bioinf..

[49] Baptista A.
M., Soares C. M. (2001). Some Theoretical
and Computational Aspects of the Inclusion
of Proton Isomerism in the Protonation Equilibrium of Proteins. J. Phys. Chem. B.

[50] Reis P. B. P. S., Vila-Viçosa D., Rocchia W., Machuqueiro M. (2020). PypKa: A Flexible
Python Module for Poisson–Boltzmann-Based pKa Calculations. J. Chem. Inf. Model..

[51] Reis P. B. P. S., Clevert D.-A., Machuqueiro M. (2021). pKPDB: a protein
data bank extension
database of p Ka and pI theoretical values. Bioinformatics.

[52] Reis P. B. P. S., Clevert D.-A., Machuqueiro M. (2024). PypKa server:
online p K a predictions
and biomolecular structure preparation with precomputed data from
PDB and AlphaFold DB. Nucleic Acids Res..

[53] Schrödinger, LLC The PyMOL Molecular Graphics System, Version 3.1. 2025.

[54] Williams, T. ; Kelley, C. Gnuplot 6.0: An Interactive Plotting Program, 2025 http://gnuplot.sourceforge.net/.

[55] Maisuradze G. G., Liwo A., Scheraga H. A. (2010). Relation
between Free Energy Landscapes
of Proteins and Dynamics. J. Chem. Theory Comput..

[56] Grosse-Holz F., Kelly S., Blaskowski S., Kaschani F., Kaiser M., van der Hoorn R. A. (2018). The transcriptome,
extracellular proteome and active
secretome of agroinfiltrated *N. benthamiana* uncover a large, diverse protease repertoire. Plant Biotechnol. J..

[57] Coculo D., Del Corpo D., Martínez M. O., Vera P., Piro G., De Caroli M., Lionetti V. (2023). Arabidopsis subtilases promote defense-related
pectin methylesterase activity and robust immune responses to botrytis
infection. Plant Physiol. Biochem..

[58] Schaller A., Stintzi A., Rivas S., Serrano I., Chichkova N. V., Vartapetian A. B., Martínez D., Guiamét J. J., Sueldo D. J., van der
Hoorn R. A. L., Ramírez V., Vera P. (2018). From structure to function
- a family portrait of plant subtilases. New
Phytol..

[59] Ottmann C., Rose R., Huttenlocher F., Cedzich A., Hauske P., Kaiser M., Huber R., Schaller A. (2009). Structural basis for
Ca2.-independence and activation by homodimerization of tomato subtilase
3. Proc. Natl. Acad. Sci. U.S.A..

[60] Cedzich A., Huttenlocher F., Kuhn B. M., Pfannstiel J., Gabler L., Stintzi A., Schaller A. (2009). The Protease-associated
Domain and C-terminal Extension Are Required for Zymogen Processing,
Sorting within the Secretory Pathway, and Activity of Tomato Subtilase
3 (SlSBT3). J. Biol. Chem..

