# Supplementary material for: In Silico Structural Analysis of the Grapevine Serine Protease VviSBT4.19 Involved in Defense against P. viticola
Source: J Agric Food Chem. 2025 Dec 24;74(1):1724–32. doi: 10.1021/acs.jafc.5c11183 (PMC12818760; doi:10.1021/acs.jafc.5c11183)
Supplement: Supplementary file 1 [file jf5c11183_si_001.pdf]

# Supporting Information: In silico structural analysis of the grapevine serine protease VviSBT4.19 involved in defense against P. viticola

Filipe E. P. Rodrigues,[†,§] Sara G. F. Ferreira,[†,§] Catarina Paiva-Silva,[‡] Andreia Figueiredo,[¶] Rita B. Santos,[*,‡] and Miguel Machuqueiro[*,†]

†*BioISI – Instituto de Biossistemas e Ciências Integrativas, Departamento de Química e Bioquímica, Faculdade de Ciências, Universidade de Lisboa, 1749-016, Lisboa, Portugal*

‡*Grapevine Pathogen Systems Lab, BioISI – Instituto de Biossistemas e Ciências Integrativas, Faculdade de Ciências, Universidade de Lisboa, 1749-016, Lisboa, Portugal*

¶*Grapevine Pathogen Systems Lab,BioISI – Instituto de Biossistemas e Ciências Integrativas, Faculdade de Ciências, Universidade de Lisboa, 1749-016, Lisboa, Portugal*

§*Both authors contributed equally*

E-mail: absantos@ciencias.ulisboa.pt; machuque@ciencias.ulisboa.pt

Phone: +351-21-7500112

Table S1: List of primers used for cDNA synthesis, cloning, and site-directed mutagenesis

| | Primer sequences (5'–3') | Ta (°C) |
|---|---|---|
| Primers for ORF isolation | 5'–aataaagttggaatgtgcatagcttaccttcta–3';<br>5'–gaaagctgggtgtgggagggctaatacaac–3' | 60 |
| Gateway cloning | 5'–ggggacaagtttgtacaaaaaagcaggcttaatgtgcatagcttaccttctaatggca–3'<br>5'–ggggaccactttgtacaagaaagctgggtttgggagggctaatacaacaatggg–3' | 60 |
| Mutagenesis | 5'–ttccgggacgaacatgtcctgcc–3'<br>5'–attatattgtatagcacttctcgattatc–3' | 65 |

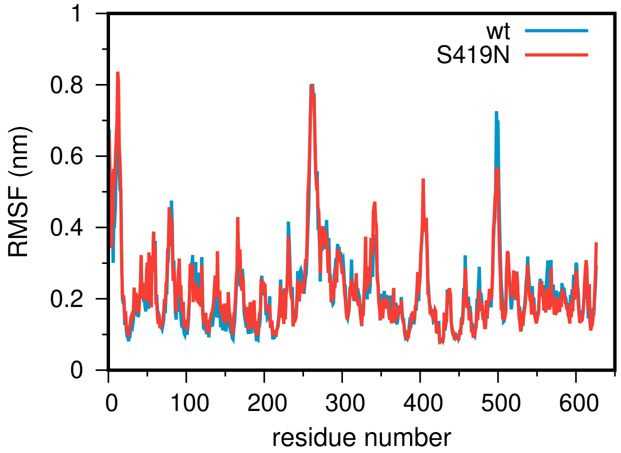


Figure S1: Residue-wise root mean square fluctuations (RMSF) of the wild-type (wt) and S419N mutant serine protease. RMSF values are shown as a function of residue number, with the wt (blue) and the S419N mutant (red) depicted for comparison.

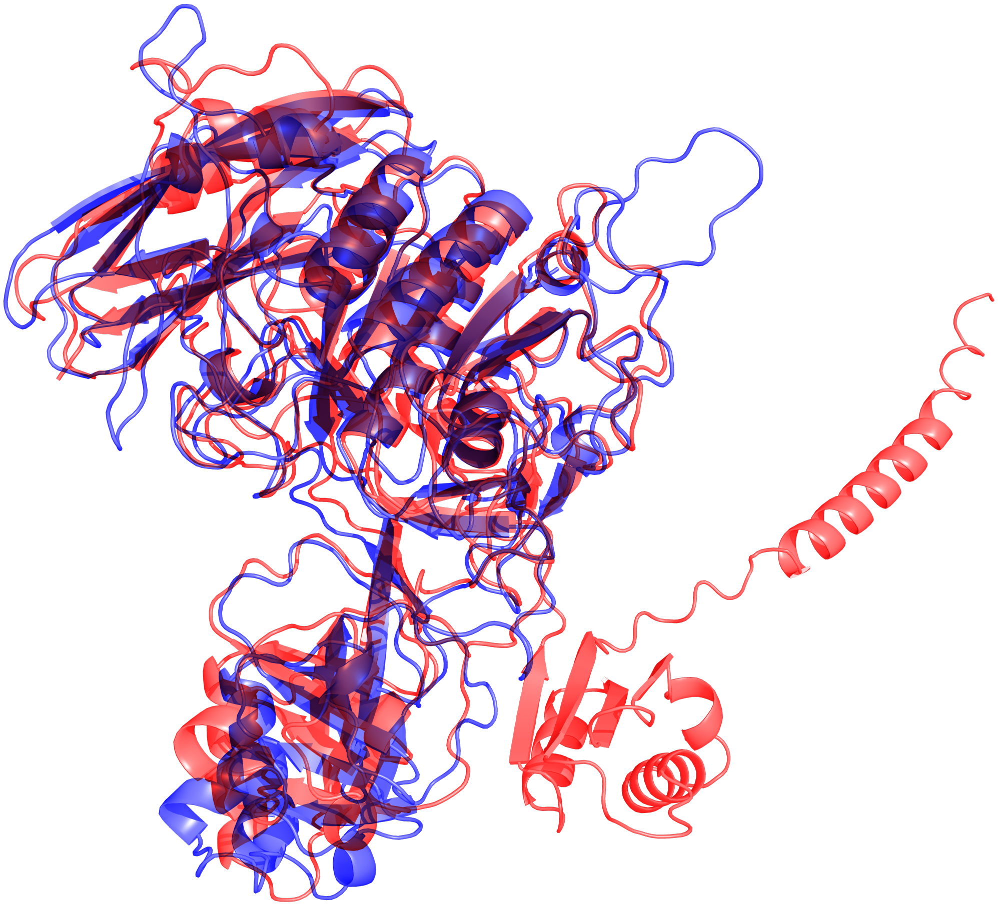

Figure S2: Structural comparison of the models generated by AlphaFold (red) and Modeller (blue). Both structures are displayed as semi-transparent cartoons and are superimposed from residue 121 to 746, emphasizing their structural similarity (RMSD of 1.2 Å). The AlphaFold model includes residues 1–120 (unaligned red region at the bottom right), while this segment is absent in the Modeller model. This segment corresponds to the I9 inhibitory domain, which must be cleaved for protein activation.

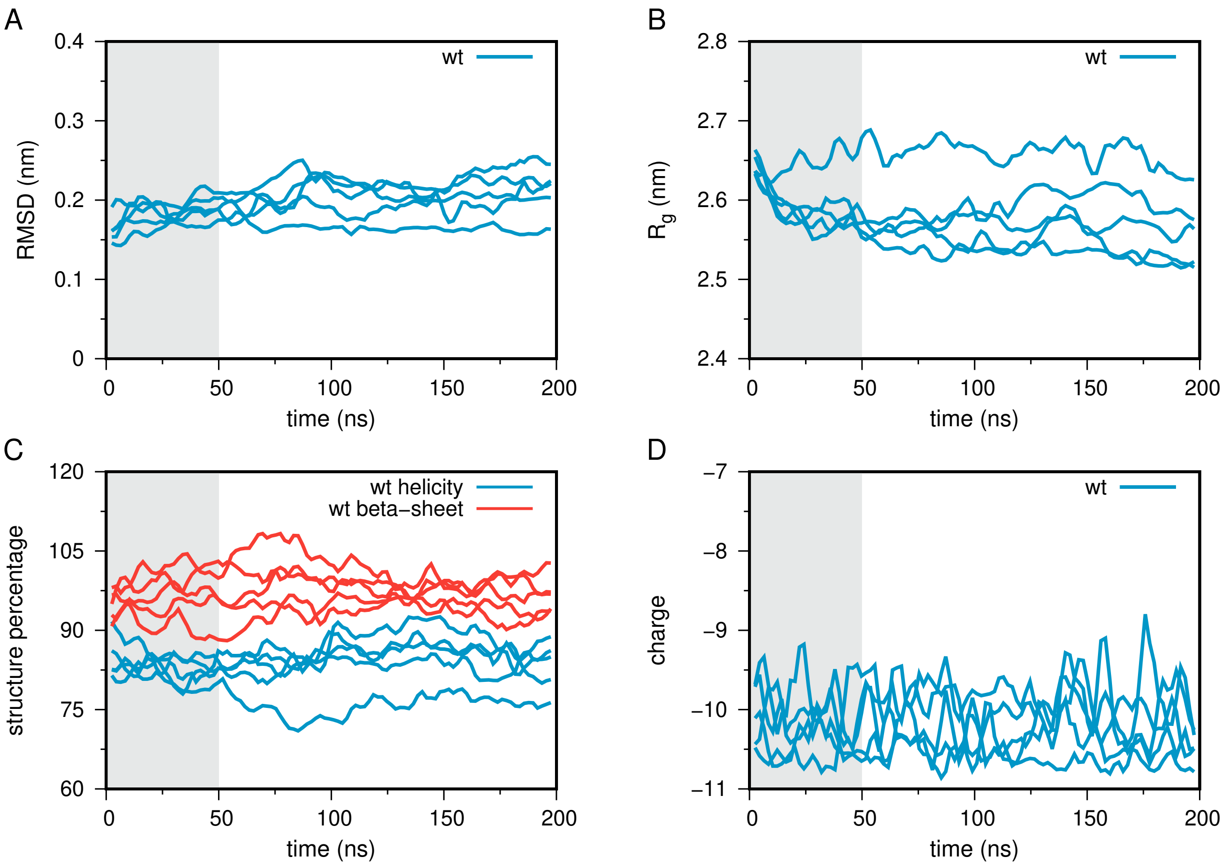


Figure S3: Time evolution of the root mean square deviation (RMSD) (a), radius of gyration ($R_g$) (b), secondary structure content (helicity and beta-sheet) (c), and total charge (d) of the *wt* serine protease. The secondary structure content is presented as a percentage relative to the corresponding content in the initial structure obtained from the homology modeling. A sliding window average (5 ns) was applied to smooth fast fluctuations of these properties. The five replicates are shown.

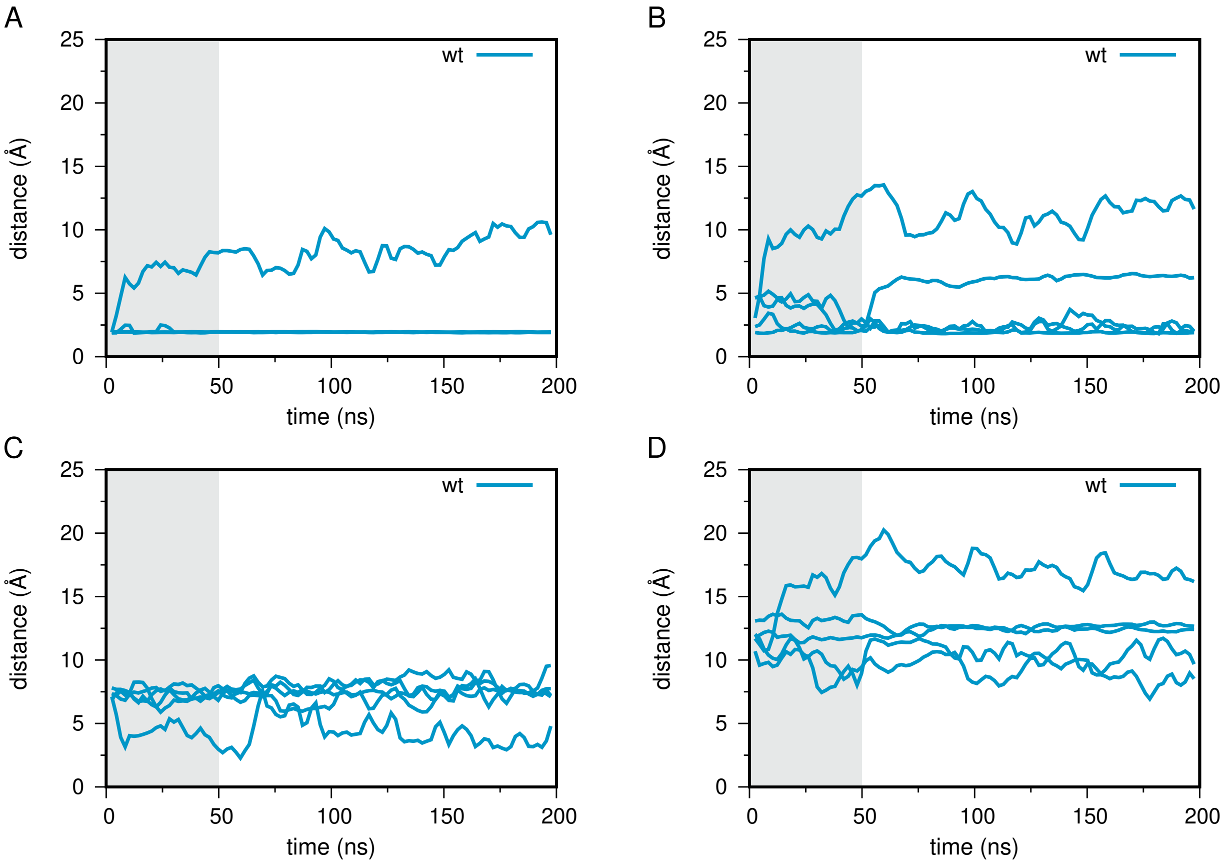


Figure S4: Time evolution of the distances between the residues that can form catalytic triads in the *wt* serine protease. The distances between the catalytic His93 and Asp29 (a), Ser419 (b), Glu91 (c), and Ser116 (d) are shown. A sliding average of 5 ns was applied to smooth fast fluctuations. The five replicates are shown.

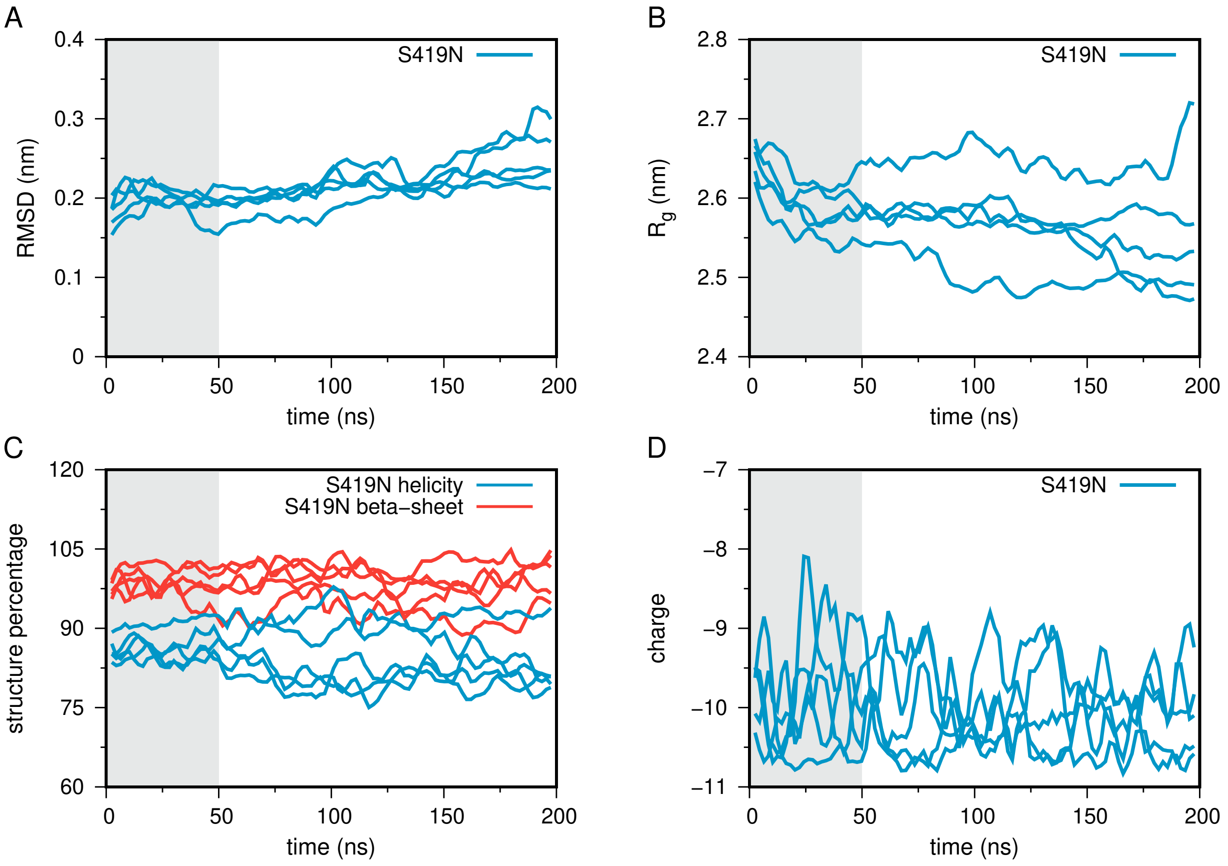


Figure S5: Time evolution of the RMSD (a), $R_g$ (b), helicity and beta-sheet content (c), and total charge (d) for the S419N mutated serine protease. For the secondary structure content, the values are shown as percentages relative to the secondary structure content of the template used for homology modeling (PDB: 4YN3). $\alpha$-helices are represented in blue, and $\beta$-strands are represented in red. A sliding average of 5 ns was applied to smooth fast fluctuations. The five replicates are shown.

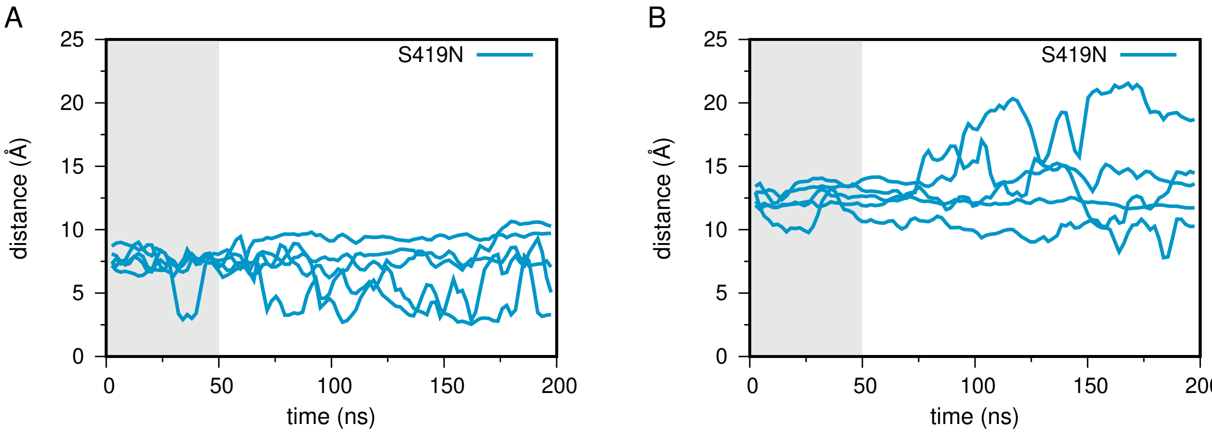


Figure S6: Time evolution of the distances between the residues that can still form a catalytic triad in the S419N mutated serine protease. The distances between His93 and Glu91 (a) or Ser416 (b) are depicted. A sliding average of 5 ns was applied to smooth fast fluctuations. The five replicates are shown.
